# Observation on efficacy and underlying mechanism of cheek acupuncture on ovulation induction for infertile women with PCOS: Case series

**DOI:** 10.1097/MD.0000000000037370

**Published:** 2024-03-08

**Authors:** Yi Yang, Lihua Jin, Shasha Xu, Huijun Ye, Xi Luo, Ruilan Li, Yuebing Yue

**Affiliations:** a Department of Obstetrics and Gynecology, The Second Affiliated Hospital of Zhejiang Chinese Medical University, Xinhua Hospital of Zhejiang Province, Hangzhou, China.

**Keywords:** cheek acupuncture therapy, HPOaxis, mechanism of action, PCOS

## Abstract

**Rationale:**

Polycystic ovary syndrome (PCOS) is the most common reproductive endocrine disorder among women of childbearing age and is the primary cause of anovulatory infertility, accounting for 70% to 80% of cases. Ovulation induction is the main treatment approach for infertile patients with PCOS. Commonly utilized medications for this purpose are clomiphene citrate (CC) and letrozole (LE). Clomiphene citrate administration results in an ovulation rate ranging from 60% to 85%, while the pregnancy rate is limited to 35% to 40%, and a further reduction is observed in live birth rates. Letrozole demonstrates a slightly higher pregnancy rate and live birth rate compared to clomiphene citrate, although challenges persist in terms of longer stimulation cycles, multiple pregnancies, and the risk of ovarian hyperstimulation syndrome (OHSS). Clinical reports indicate that acupuncture therapy shows promising efficacy in treating patients with PCOS-related infertility, despite a partially unclear understanding of its underlying mechanisms.

**Patient concerns:**

In this study, one patient did not achieve pregnancy despite more than a year of ovulation induction using clomiphene citrate and letrozole. However, after 3 months of receiving cheek acupuncture therapy, she successfully conceived and gave birth to a liveborn baby. Another patient achieved natural conception and live birth after 2 months of exclusive cheek acupuncture therapy.

**Diagnosis:**

PCOS.

**Interventions:**

Cheek acupuncture therapy.

**Outcomes:**

Both of them successfully conceived and gave birth to a liveborn baby.

**Lessons:**

These findings suggest that cheek acupuncture therapy can effectively stimulate follicle development and ovulation, potentially improving endometrial receptivity. According to holographic theory, there is a biologically holographic model within the cheek region that shares a homology with the human body structure. This model provides an explanation for the regulatory effects of cheek acupuncture point stimulation on the Hypothalamic-Pituitary-Ovarian axis (HPO), which subsequently influences follicle development and ovulation in patients. Consequently, when cheek acupuncture therapy is applied alone or in combination with ovulation induction medication, patients have the ability to achieve successful pregnancy and experience a smooth delivery.

**Highlights**

Ovulation induction medications are frequently employed to tackle infertility in PCOS patients. However, this approach is associated with a low rate of pregnancy and live birth, as well as the risk of such complications as ovarian hyperstimulation syndrome (OHSS). Consequently, it is imperative to explore novel approaches for managing infertility in PCOS. Acupuncture has been extensively used in China for the treatment of PCOS-associated infertility for many years, demonstrating favorable therapeutic effects. This article presents a novel treatment approach for PCOS utilizing cheek acupuncture therapy. Cheek acupuncture therapy is a specialized branch of micro-needle systems that incorporates holographic theory. By leveraging the precise anatomical structure of the cranial bones, 16 standardized acupuncture points are precisely identified on bilateral cheeks. This method involves the targeted insertion of needles into these acupuncture points to treat various ailments. Holographic theory suggests that stimulation of the acupuncture points on the cheeks can regulate the hypothalamic-pituitary-ovarian (HPO) axis and the autonomic nervous system. This, in turn, modulates follicle development and ovulation in patients.

## 1. Introduction

PCOS is the prevailing reproductive endocrine disorder among women of reproductive age,^[1]^ accounting for 70% to 80% of cases.^[2]^ Ovulation induction is the main treatment approach for infertile patients with PCOS,^[3,4]^ although challenges persist in terms of longer stimulation cycles, multiple pregnancies, and the risk of ovarian hyperstimulation syndrome (OHSS).^[5]^ Clinical reports indicate that acupuncture therapy shows promising efficacy in treating patients with PCOS-related infertility,^[6,7]^ despite a partially unclear understanding of its underlying mechanisms. PCOS is characterized by neuroendocrine dysfunction, particularly dysfunction in the hypothalamic-pituitary-ovarian (HPO) axis, which is frequently observed in patients with PCOS.^[8]^ Altered pulsatile secretion of gonadotropin-releasing hormone (GnRH) by the hypothalamus results in increased luteinizing hormone (LH) secretion and insufficient follicle-stimulating hormone (FSH) secretion from the pituitary gland. This decreases aromatase levels in granulosa cells, impeding the conversion of testosterone (T) to estrogen. Subsequently, elevated LH and androgen levels arise, causing stagnation in follicular development and anovulation. The diagnosis of PCOS is based on the Rotterdam criteria,^[9]^ which were developed jointly by the European Society of Human Reproduction and Embryology and the American Society for Reproductive Medicine in January 2003. Additionally, the Chinese Society of Obstetrics and Gynecology Endocrinology Group and Guideline Expert Group have issued the *Chinese Guidelines for Diagnosis and Treatment of Polycystic Ovary Syndrome*.^[10]^ In order to meet the diagnostic criteria, a minimum of 2 out of the following 3 criteria must be satisfied: oligoovulation or anovulation, clinical or biochemical hyperandrogenism, and ultrasonography indicating the presence of ≥ 12 small follicles with a diameter of 2 to 9 mm in both ovaries and/or ovarian volume ≥ 10 mL. CC works as a selective estrogen receptor modulator by competitively attaching to nuclear estrogen receptors. As the negative feedback of estrogen is reduced, secretion of gonadotropin hormones increases, inducing ovarian follicular growth.^[11]^ CC, however, has a relatively long half-life of around 14 days and exerts antiestrogenic effects in the periphery. Consequently, it suppresses the growth of the uterine endometrium during the late phase following ovulation. This also explains why CC administration is associated with a higher ovulation rate but a lower pregnancy rate.^[3]^ LE is a potent and specific aromatase inhibitor that hinders the conversion of androgens into estrogens. Moreover, reduced estrogen levels decrease negative feedback to the hypothalamus and stimulate greater secretion of FSH, thus facilitating ovulation. Another mechanism through which LE promotes ovulation involves a transient elevation in intraovarian androgens, thereby enhancing follicular sensitivity to FSH.^[12]^ LE demonstrates superior pregnancy and live birth rates compared with CC, while also providing a certain degree of reduction in the risk of multiple pregnancies.^[13]^ Nevertheless, it is not without limitations, particularly in relation to ovarian hyperstimulation and drug resistance.^[14]^ Consequently, an escalating number of patients in China and several other Asian countries are turning to traditional Chinese herbal medicine or acupuncture as treatment modalities for PCOS.^[15]^ Reports indicate that acupuncture is a significant intervention for improving menstrual cycles, reducing body mass index (BMI), as well as total testosterone and LH levels in women diagnosed with PCOS.^[16]^ Following a 3-month treatment period, Dong’s acupuncture demonstrated a significant reduction in LH levels and the LH/FSH ratio, while also improving the number of polycystic ovaries.^[17]^ Zhuo et al^[18]^ employed the “Tiao Ren Tong Du” acupuncture technique for treating PCOS and discovered its potential for restoring regular menstrual cycles, enhancing endometrial thickness, fostering follicle development and maturation, and effectively decreasing LH levels.

Cheek acupuncture therapy is a specialized branch of the micro-needle system that utilizes holographic theory to treat diseases by targeting specific acupoints on the cheek.^[19]^ It has demonstrated exceptional efficacy in managing pain-related ailments. In comparison to body acupuncture, cheek acupuncture therapy offers swift analgesic effects while also exerting systemic regulatory effects.^[20]^ Furthermore, it has exhibited considerable therapeutic effects in the management of internal medical conditions.

In this report, we documented the application of cheek needle therapy for treating infertility associated with polycystic ovary syndrome, resulting in successful conception and delivery in both patients. The potential mechanism of action of this new technique is also discussed.

The Second Affiliated Hospital of Zhejiang Chinese Medical University Ethics Commission for human research sanctioned the study, ensuring the ethical standards and integrity of the research were maintained, and the patient gave informed consent.

## 2. Case description

### 2.1. Case 1

**Figure 1.**
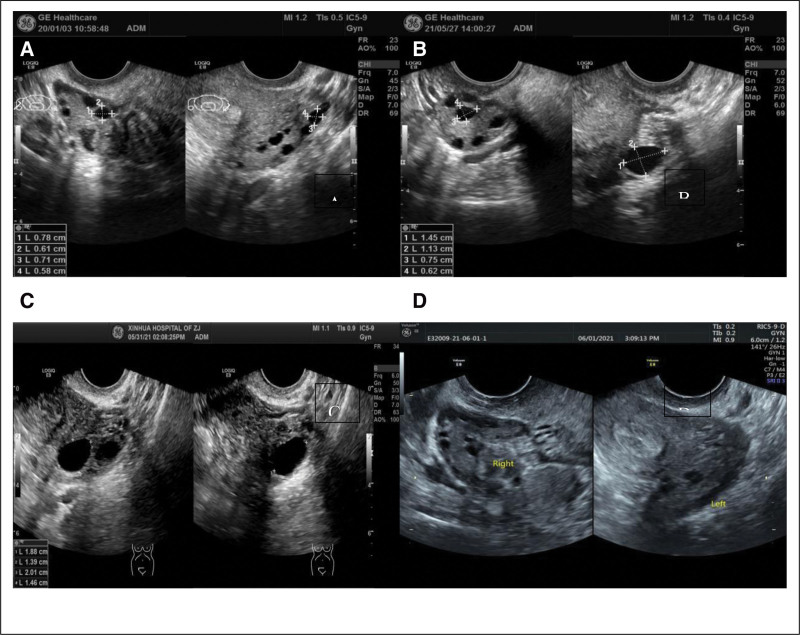
illustrates the comparison of ovarian polycystic changes in Case 1 before and after treatment. Arrows are used to indicate the follicle situation. (A) presents the follicle condition prior to treatment. (B) depicts the follicle development on the 13th day of the menstrual cycle. (C) shows the follicle development on the 17th day of the menstrual cycle, and (D) represents the post-discharge situation of the follicle on the 18th day of the menstrual cycle.

**Figure 2.**
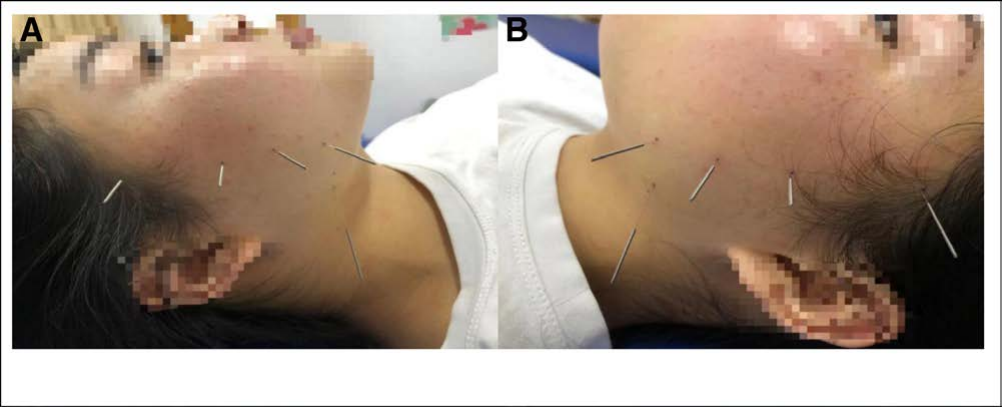
illustrates the specific acupoints utilized for cheek acupuncture treatment in Case 1. (A) denotes the acupoints applied on the right cheek, whereas (B) represents the acupoints employed on the left cheek.

**Figure 3.**
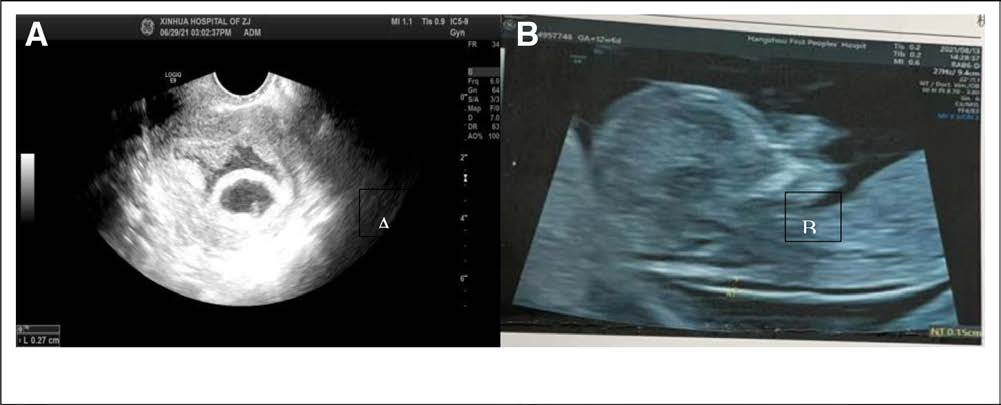
presents ultrasound images of the patient in Case 1 throughout pregnancy. (A) depicts the initial confirmation of intrauterine pregnancy, while (B) illustrates the fetal nuchal translucency ultrasound.

A 32-year-old female patient presented at our hospital on January 3, 2021, seeking assistance with fertility issues as she had been unable to conceive for 2 years despite not using contraception. Before coming to our hospital, the patient had previously received prescriptions for medications like clomiphene and letrozole from another medical facility for 1 year to stimulate ovulation. The patient’s menstrual cycle is 30 to 35 days, and the duration of the period is 6 to 7 days. The patient experiences a moderate menstrual flow without significant cramping. The patient’s measurements are as follows: height – 154 cm, weight – 65 kg, BMI – 27.4. Blood tests revealed the following hormone levels: FSH – 3.8 IU/L, LH – 6.14 IU/L, estradiol (E2) – 189.8 pmol/L, and testosterone (T) – 2.02 nmol/L. Ultrasound examination revealed that the left ovary measures 3.5 × 3.2 cm, while the right ovary measures 3.1 × 2.6 cm. Additionally, multiple small areas with cystic hypoechogenicity were observed in both ovaries. The uterus is retroverted and of normal size. The myometrium exhibits homogenous echoes. The endometrium is centrally positioned, and the uterine cavity presents clear visualization. Bilaterally, the endometrium measures approximately 0.5 cm in thickness as depicted (Fig. 1). The hysterosalpingography results confirm the bilateral patency of the fallopian tubes, and the male partner’s semen analysis shows mostly normal findings. LE was administered from the 5th day of the menstrual cycle to induce ovulation. The medication was taken continuously for 5 days. Ultrasonography revealed the presence of dominant follicles, indicating successful ovulation. Timing for sexual intercourse was guided based on ultrasonography and serum hormone tests, including estradiol (E2), follicle-stimulating hormone (FSH), luteinizing hormone (LH), and progesterone (Prog). This protocol was implemented for 3 consecutive months. The patient experienced sensitivity during ovulation, manifesting as significant abdominal pain (VAS score of 4). Despite this, pregnancy did not occur. Cheek acupuncture therapy was introduced to the LE (leutinizing hormone) protocol for promoting ovulation after a one-month break (Fig. 2). Cheek acupuncture therapy commenced on the 5th day of the menstrual cycle and continued every other day until ovulation was completed. Ultrasonic monitoring revealed the presence of dominant follicles, confirming successful ovulation.During the second month of treatment, follicle monitoring was conducted. On May 27, 2021, ultrasound examination showed the presence of 2 larger follicles in the left ovary, measuring 1.4 × 1.1 cm and 1.3 × 1.1 cm in diameter. Ultrasound on May 29, 2021 revealed the presence of a dominant follicle in the left ovary, measuring 2.0 × 1.4 cm in diameter. Ultrasound examination on May 30, 2021 detected 2 mature follicles in the left ovary, measuring 2.0 × 1.4 cm and 1.8 × 1.4 cm in diameter. The follicles exhibited thin walls and good echogenicity. Ultrasound examination on June 1, 2021 revealed the absence of visible follicles in both ovaries (Fig. 1). On June 13, 2021, the patient’s serum beta-HCG level was measured at 79.4 IU/L, along with E2 (estradiol) level of 1067 pmol/L and Prog (progesterone) level of 145.4 nmol/L, which indicated a potential pregnancy. Follow-up examination on June 24, 2021, showed a serum beta-HCG level of 13812 IU/L, along with E2 (estradiol) level of 2324 pmol/L and Prog (progesterone) level of 118.8 nmol/L. On June 29, 2021, ultrasound examination detected a gestational sac measuring 2.3 × 1.3 cm within the uterus. The embryo measured approximately 0.3 cm in length and exhibited visible primitive cardiac activity. Subsequent prenatal examinations conducted at our hospital revealed satisfactory fetal growth. On August 13, 2021, a nuchal translucency ultrasound examination indicated a measurement of 0.13 cm (Fig. 3). On October 22, 2021, three-dimensional ultrasound showed normal fetal growth and development, with no significant abnormalities detected during screening. The patient underwent a cesarean section delivery on February 15, 2022, at 39 weeks of gestation. The delivery resulted in the birth of a live female infant weighing 3600 kg. The infant received an Apgar score of 10 at birth. The infant was monitored for 1 year and displayed normal growth and development.

### 2.2. Case 2

**Figure 4.**
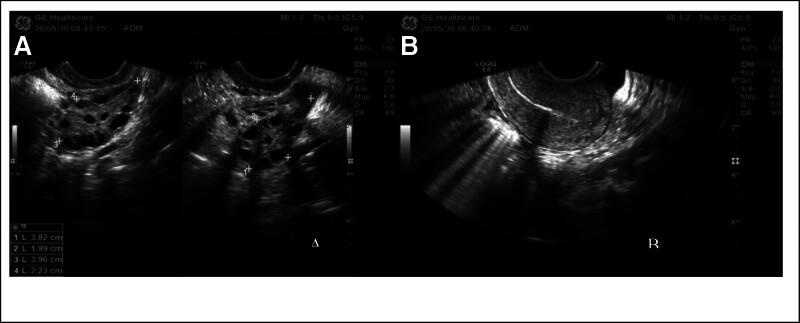
illustrates the comparison of ovarian changes in Case 2 prior to polycystic ovary treatment. Arrows indicate the follicular conditions, (A) represents bilateral ovarian follicles, (B) portrays the conditions of the uterus and endometrium.

**Figure 5.**
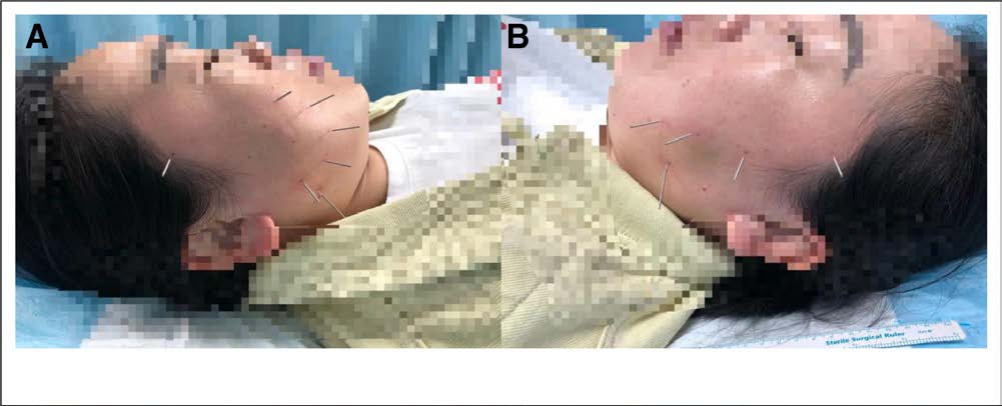
presents the acupoint locations used for cheek acupuncture treatment in Case 2. (A) indicates the usage of acupoints on the right cheek. (B) represents the utilization of acupoints on the left cheek.

**Figure 6.**
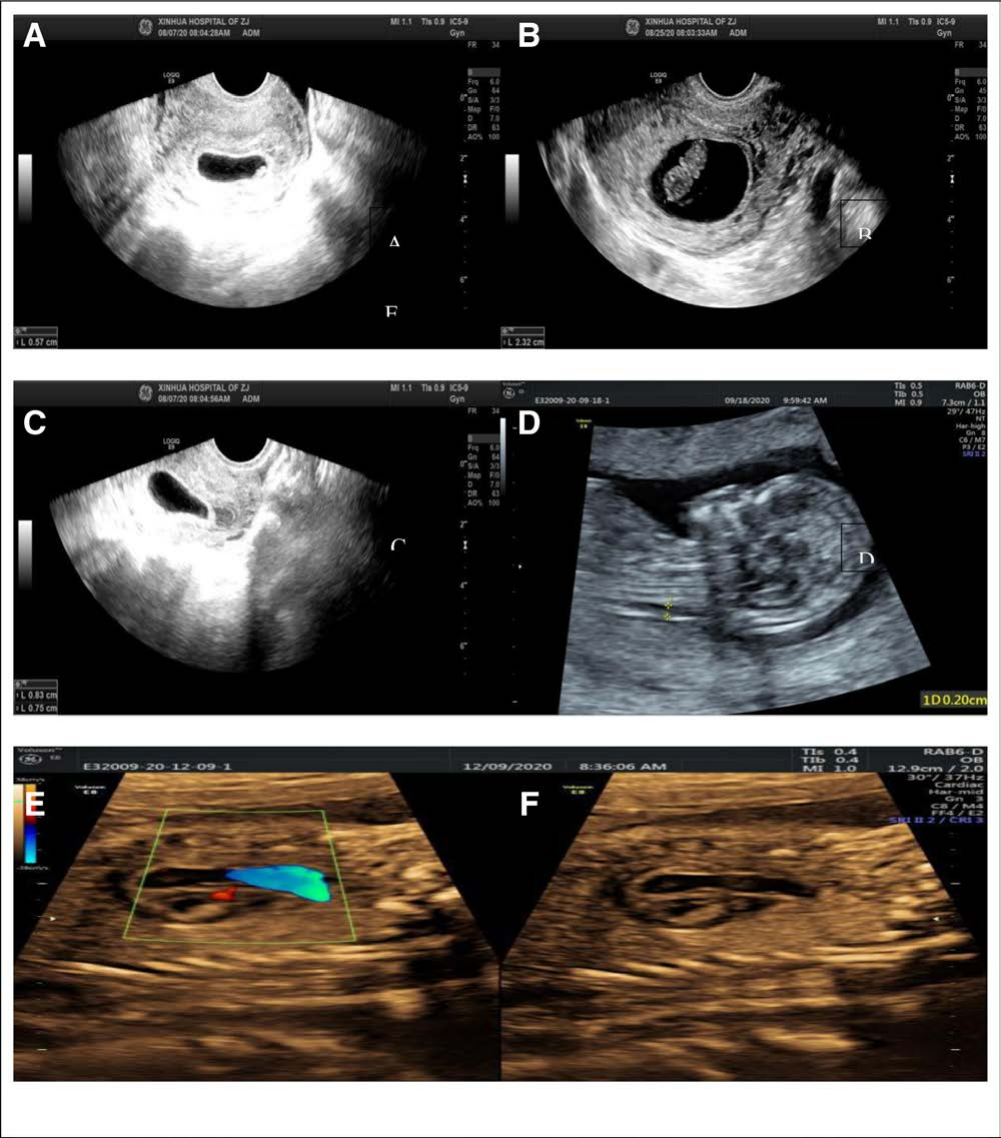
depicts ultrasound images at different stages of pregnancy: (A) confirms the initial intrauterine pregnancy, (B) displays the presence of a fluid-filled hypoechoic region in early pregnancy that subsequently disappears, (C) represents the follow-up ultrasound showing normal embryo growth, (D) demonstrates fetal nuchal translucency, (E) presents three-dimensional ultrasound images of the fetus.

On May 30, 2020, a 23-year-old patient visited our hospital after giving birth to a child for 2 years, reporting the resumption of menstruation following cessation of breastfeeding. The patient had a menstrual cycle that ranged from 27 to 40 days, with a duration of 5 days. Before seeking medical consultation at our hospital, the patient had been attempting to conceive without success for a period of 1 year and 5 months, and had not utilized any contraceptive methods. The patient was diagnosed with polycystic ovary syndrome (PCOS) 4 years ago and achieved a successful childbirth through induced ovulation treatment in January 2018, resulting in the birth of a live infant. The patient has a height of 170 cm, weight of 75 kg, and a BMI of 25.95. Blood tests indicated the following hormone levels: follicle-stimulating hormone (FSH) at 7.6 IU/L, luteinizing hormone (LH) at 15.88 IU/L, estradiol (E2) at 198.2 pmol/L, and testosterone (T) at 1.82 nmol/L. The ultrasound examination revealed a size of 2.7 × 2.6 × 2.4 cm for the left ovary and 3.0 × 2.9 × 2.8 cm for the right ovary. Within both ovaries, numerous small cystic hypoechoic areas were observed. The largest cyst measured 1.0 cm in diameter. The uterus exhibited retroversion and had a normal size, along with uniform myometrial echoes. The centrally located endometrium had a thickness of approximately 0.17 cm on the posterior side (Fig. 4). The hysterosalpingography using iodine oil reveals that both fallopian tubes are unobstructed, and the male partner’s semen analysis shows essentially normal results. Due to the patient’s refusal of conventional medication for ovulation induction, cheek acupuncture therapy was utilized as an alternative to stimulate ovulation (Fig. 5). Acupuncture treatment commenced on the 5th day of the menstrual cycle and was administered every other day until ovulation completed. Ultrasound monitoring indicated favorable growth of dominant follicles. Sexual intercourse was guided based on the ultrasound findings and serum hormone tests including E2, FSH, LH, and Prog. Pregnancy was indicated on July 27, 2020, when the patient’s serum β-HCG level was monitored and measured to be 2548 IU/L, E2 level was 1334 pmol/L, and Prog level was 45.55 nmol/L. The patient presented with complaints of mild lower abdominal pain and scanty coffee-colored vaginal bleeding on August 7, 2021.The ultrasound examination detected a regularly shaped gestational sac measuring 3.0 × 1.0 cm within the uterus. An anechoic fluid-filled region measuring 0.8 × 0.8 cm was visualized between the gestational sac and the uterine wall. The embryo exhibited a length of approximately 0.6 cm and demonstrated visible evidence of primitive cardiac activity. The patient was prescribed oral administration of 40 mg progesterone. A follow-up ultrasound conducted 2 weeks later revealed a regularly shaped gestational sac measuring 4.6 × 2.8 cm inside the uterus. The crown-rump length of the embryo measured 2.3 cm, and visible evidence of primitive cardiac activity was observed. The patient had regular prenatal examinations at our hospital, and the fetus exhibited satisfactory growth. Nuchal translucency ultrasound on September 18, 2021 revealed a measurement of 0.21 cm. A 3D ultrasound conducted on December 9, 2021 showed normal fetal growth without any significant anomalies observed (Fig. 6).

### 2.3. Acupuncture manipulation

Both patients received treatment from the same acupuncturist for the entire duration of their acupuncture therapy, ensuring consistent acupoint selection and treatment procedures. The acupuncture points and procedures adhered strictly to the guidelines outlined in “Cheek Acupuncture Therapy,” ensuring accurate placement of acupoints and appropriate needle insertion depths as prescribed. Each patient receives treatment every other day, with each session lasting for 30 minutes. Treatments were initiated on the 5th day of the menstrual cycle and continued until ovulation, with treatments administered every other day. Table 1 displays the standardized acupoint locations and their corresponding therapeutic indications utilized in this study. Incheek acupuncture therapy, acupoint selection is primarily guided by the theory of biocybernetics. The CA-1 acupoint is positioned on the cheek, aligning with the location of the hypothalamus in the head. The CA-8 acupoint is situated within the central pelvic cavity, preceding S1 to S5, and corresponds to the anatomical positioning of the uterus and ovaries in the pelvic region. It is located 0.5 inch above the anterior lower angle of the jaw. Additionally, according to the theory of the 3 energizers in cheek acupuncture therapy, acupoints in the upper, middle, and lower energizer are chosen to complement the acupoints in the head and sacral regions.

**Table 1:** Standard acupoints of cheek acupuncture, their main applications and acupoints used in the present study.

Name	Orientation	Application
Head point CA-1	1 inch above the upper edge of the middle point of the zygomatic arch	Headache, dizziness, toothache, insomnia, stress, anxiety, depression, stroke, etc.
Upper energizer point CA-2	The cross of the posterior coronoid of the mandible and the lower edge of the zygomatic arch	Headache, cervical pain, chest pain, chest tightness, breast swelling and pain, tachycardia, arrhythmia, asthma, etc.
Middle energizer point CA-3	The middle point of the connecting line between the upper and lower energizer acupoints	Stomach cramp, acute/chronic gastritis, heartburn with acidity
Lower energizer point CA-4	Inner side of the anterior margin of the mandibular angle	Abdominal bloating and pain, colitis, dysmenorrhea, pelvic inflammatory disease, menstrual irregularities, leukorrhea, gynecological disease
Sacral point CA-8	0.5 inch to the anterior & superior angle of the mandible	Sacrospinous muscle strain, lower back pain in women, injuries of sacroiliac ligament, bedwetting, prostatitis, etc.

## 3. Discussion

Commonly used medications for ovulation induction in PCOS patients include clomiphene citrate and letrozole. Although these medications have demonstrated good efficacy in promoting ovulation, they are also linked to lower rates of pregnancy and live birth as well as an increased risk of ovarian hyperstimulation syndrome. Both patients in this study received ovulation induction treatment through the application of cheek acupuncture therapy. Throughout the acupuncture treatment, follicle development was observed, leading to successful ovulation, conception, and live births in both patients, ultimately resulting in positive outcomes. This, to some extent, suggests that cheek acupuncture therapy has the potential to promote follicular development and induce ovulation. Nevertheless, additional extensive experimental research is required to establish the reliability of the therapeutic effects of cheek acupuncture therapy and to investigate its underlying mechanism.

As a microsystem branch of acupuncture, Cheek acupuncture therapy utilizes holographic theory to treat diseases by targeting specific acupuncture points on the cheeks.^[19]^ This modality has demonstrated notable efficacy in treating painful conditions and exerts systemic regulatory effects.^[20]^ Experimental studies have demonstrated that cheek acupuncture treatment for rheumatoid arthritis induces an immediate analgesic effect by upregulating Met-enkephalin and cholecystokinin octapeptide levels in the cerebrospinal fluid.^[21]^ Besides its application in pain management, cheek acupuncture therapy is also used for treating other various diseases. For instance, Dr LIU et al demonstrated that cheek acupuncture therapy can effectively enhance hearing status and alleviate tinnitus in patients with hearing impairment following chemotherapy for nasopharyngeal carcinoma.^[22]^ The utilization of cheek acupuncture therapy in managing obstructive sleep apnea-hypopnea syndrome (OSAHS) was documented by Dr YE et al Cheek acupuncture therapy has the capacity to directly improve respiratory pauses and hypoxemia associated with upper airway obstruction, as well as alleviate metabolic abnormalities in glucose and lipid metabolism that arise from obesity and overweight conditions.^[23]^ In a study by Dr WU et al, it was reported that combining cheek acupuncture with Danhong injection enhances therapeutic efficacy, reduces the frequency of angina attacks, decreases levels of serum C-reactive protein expression, and improves coronary artery blood flow reserve capacity in patients diagnosed with X syndrome of the heart.^[24]^ Hence, there is compelling evidence to support the notion that cheek acupuncture is not only effective in the management of painful conditions but also beneficial in the treatment of diverse ailments.

Our experiment yielded positive therapeutic effects through the utilization of cheek acupuncture therapy to stimulate ovulation. In this study, we initially utilized the theory of biologic holography, which recognizes that the hypothalamus is situated below the hypothalamic sulcus, forming the lower wall of the third ventricle with an undefined boundary. The hypothalamus extends downward and is connected to the pituitary stalk. The acupoint CA-1 on the head was chosen, specifically located1 inch above the upper edge of the middle point of the zygomatic arch. To regulate the HPO axis, we targeted the sacral acupoint CA-8, which is located 0.5 inch to the anterior & superior angle of the mandible, corresponding to the central region of the pelvic cavity in front of S1 to S5. Subsequently, we utilized the theory of Triple Energizer, which is renowned for its potent capacity in qi transformation and channel regulation. Smooth operation of the Triple Energizer ensures unobstructed communication between internal and external, upper and lower, and surface and interior pathways. Conversely, the presence of any obstruction within the Triple Energizer can result in localized or systemic stagnation of qi circulation, impairing blood flow and giving rise to diverse health disorders. As a result, the Triple Energizer acupoint is widely acknowledged as a crucial acupuncture point for regulating organ function and promoting overall qi circulation.^[25]^ Based on this rationale, we made the decision to include bilateral Triple Energizer acupoints as part of our approach to harmonize the comprehensive qi circulation system and mitigate diverse ailments resulting from Qi stagnation. The upper burner acupoint CA-2 is positioned at the cross of the posterior coronoid of the mandible and the lower edge of the zygomatic arch. The middle burner acupoint CA-3 is located at the connecting line between the upper and lower energizer acupoints. The lower burner acupoint CA-4 is situated on the inner side of the anterior margin of the mandibular angle.

**Figure 7.**
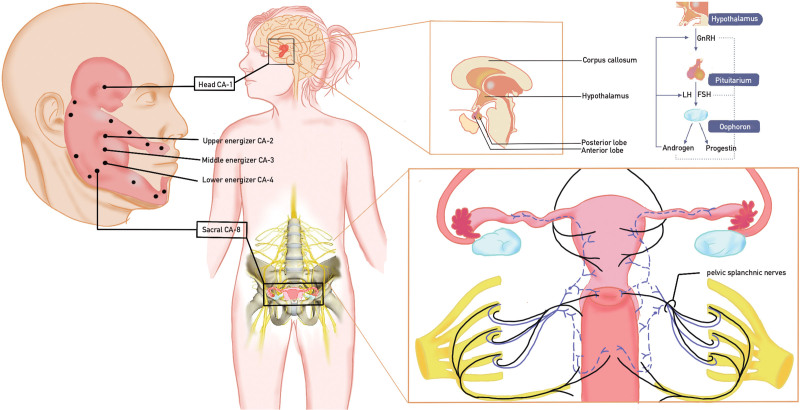
The simplified model of the mechanism of action of cheek acupuncture.

Numerous studies have examined the mechanism by which acupuncture regulates the HPO axis. Experimental evidence has demonstrated a connection between acupuncture treatment for PCOS and the stimulation of β-endorphin production in the hypothalamus. Moreover, acupuncture has been shown to modulate the autonomic nervous system and influence the release of GnRH and corticotropin-releasing hormone, thereby potentially affecting reproductive functions including LH and FSH levels.^[26]^ Animal experiments have demonstrated that the rostral medium septum (MS), diagonal band of Broca, and medial preoptic area (MPO) exhibit the highest density of GnRH neurons in the brain.^[27]^ The majority of GnRH neurons originate from the hypothalamic median eminence, where they release GnRH into the portal vein system of the pituitary gland.^[28]^ Elevated GnRH levels in the preoptic area result in an augmented secretion of LH.^[29]^ Acupuncture was administered to the head acupoint (CA-1) in 2 clinical cases. Based on the holographic theory of the facial region, the chosen acupoints were positioned in proximity to the subthalamic area of the holographic embryo. After needling CA-1, the trigeminal and facial nerves, which are distributed within this acupoint, convey signals to the sensory ganglia linked to the corresponding subthalamic area of the holographic embryo. Throughout this process, the acupuncture and nociceptive signals are conveyed and analyzed. The β-endorphin, produced as a result of acupuncture, activates its effects on the subthalamus,^[30,31]^ thereby reducing GnRH secretion^[32]^ and influencing the HPO axis. At the same time, we administered acupuncture to the sacral acupoint (CA-8). The identified CA-8 acupoint corresponds to the second sacral vertebra, whereas the sacral center of the parasympathetic nervous system is precisely located between the second and fourth sacral vertebrae. Following needle insertion into CA-8, the trigeminal and facial nerves, which are distributed within this acupoint, receive stimulation and transmit signals to the sensory ganglia associated with the corresponding sacral area of the holographic embryo, particularly the second sacral region. Throughout this process, acupuncture and nociceptive signals are transmitted and analyzed, with acupuncture-generated β-endorphin acting on the parasympathetic sacral center. Experimental evidence indicates that individuals with PCOS exhibit a reduction in the dynamic activity of the autonomic nervous system, possibly due to decreased parasympathetic activity and increased sympathetic activity.^[33]^ It is hypothesized that following acupuncture, β-endorphin targets the parasympathetic sacral center, leading to increased parasympathetic activity and subsequent reduction in testosterone levels.^[34]^ (See Fig. 7 for visualization.)The synergistic effects of both acupoints on reproductive hormones help restore endocrine hormone homeostasis in PCOS patients, consequently facilitating regular ovulation and fertility.

## 4. Conclusion

Following a 2 to 3 month course of acupuncture, both patients experienced successful ovulation and achieved pregnancy. Throughout a 2-year follow-up period, it was observed that both patients had successful pregnancies and gave birth to healthy infants. Regarding the mechanism by which cheek acupuncture promotes ovulation, we hypothesize that it involves the precise regulation of the HPO axis and the reduction of testosterone levels through holographic modeling and accurate modulation of the sacral region, which influences the hypothalamus and parasympathetic nervous system. Cheek acupuncture therapy stands out for its standardized approach, painless procedure, and convenient application, rendering it suitable for clinical utilization and broad dissemination. However, the efficacy and mechanisms of action necessitate additional validation in the form of large-scale randomized controlled trials (RCTs) and animal experiments.

## Author contributions

**Data curation:** Lihua Jin.

**Formal analysis:** Shasha Xu.

**Investigation:** Xi Luo.

**Validation:** Huijun Ye.

**Writing – original draft:** Yi Yang, Yuebing Yue.

**Writing – review & editing:** Ruilan Li, Yuebing Yue.
